# Understanding the Role of Biofilms in Acute Recurrent Tonsillitis through 3D Bioprinting of a Novel Gelatin-PEGDA Hydrogel

**DOI:** 10.3390/bioengineering11030202

**Published:** 2024-02-21

**Authors:** Oliver Denton, Yifei Wan, Laura Beattie, Téa Jack, Preston McGoldrick, Holly McAllister, Cara Mullan, Catriona M. Douglas, Wenmiao Shu

**Affiliations:** 1 Department of Biomedical Engineering, University of Strathclyde, Glasgow G1 1XQ, UK; dentonog@doctors.org.uk (O.D.);; 2 Department of Otolaryngology/ENT Surgery, NHS Greater Glasgow and Clyde, Glasgow G51 4TF, UK; 3 Department of Medicine, University of Glasgow, Glasgow G12 8QQ, UK; 4 Strathclyde Institute of Pharmacy and Biomedical Sciences, University of Strathclyde, Glasgow G4 0RE, UK

**Keywords:** bioprinting, hydrogel, gelatin, three-dimensional, bacteria, biofilm, tonsil, tonsillitis, antibiotic resistance

## Abstract

Acute recurrent tonsillitis is a chronic, biofilm-related infection that is a significant burden to patients and healthcare systems. It is often treated with repeated courses of antibiotics, which contributes to antimicrobial resistance. Studying biofilms is key to understanding this disease. In vitro modelling using 3D bioprinted hydrogels is a promising approach to achieve this. A novel gelatin-PEGDA pseudomonas fluorescens-laden bioink was developed and bioprinted in a 3D hydrogel construct fabricated using computer-aided design to mimic the tonsillar biofilm environment. The bioprinted constructs were cultured at 37 °C in lysogeny broth for 12 days. Bacterial growth was assessed by spectrophotometry. Cellular viability analysis was conducted using optical fluorescence microscopy (FDA/PI staining). A biocompatible 3D-printed bacteria-laden hydrogel construct was successfully fabricated. Bacterial growth was observed using optical fluorescence microscopy. A live/dead cellular-staining protocol demonstrated bacterial viability. Results obtained after the 12-day culture period showed higher bacterial growth in the 1% gelatin concentration construct compared to the 0% control. This study demonstrates the first use of a bacteria-laden gelatin-PEGDA hydrogel for biofabrication of a 3D-printed construct designed to model acute recurrent tonsillitis. Initiating a study with clinically relevant ex vivo tonsil bacteria will be an important next step in improving treatment of this impactful but understudied disease.

## 1. Introduction

Tonsils are secondary lymphoid tissue. They have a heterogeneous distribution of immune cells, playing a large role in monitoring and capturing pathogens to perform immune surveillance. Tonsillitis is the body’s immune and inflammatory response to bacterial and viral infection. The most common bacterial infection is group A streptococus, which is thought to account for 30% of infections. The global incidence of group A streptococcus tonsillitis is estimated to be 600-million cases per year. Tonsillitis has a significant impact on quality of life, with 35-million days lost from school or work each year in the UK [1]. As a clinical entity, it impacts an otherwise fit and healthy cohort of young patients in the prime of their education and working life. Penicillin is the antibiotic of choice for the treatment of tonsillitis. However, antibiotic treatment failure occurs in 20–40% of group A streptococcus tonsillitis. Biofilms enable bacteria to survive and tolerate both the host immune system and antimicrobial treatment. Consequently, biofilm-associated infections are often chronic and recurrent, contributing to antibiotic failure. Biofilms have been demonstrated to play a key role in the pathogenesis of various recurrent and debilitating conditions of the ear, nose, and throat, including otitis media with effusion in children and chronic rhinosinusitis.

Although biofilms have been suggested as a possible explanation for the chronicity and severity of tonsil infections, there have been very few studies investigating the presence of biofilms in tonsils. One of the few studies published thus far used light and electron microscopy on tonsil specimens excised for infections and tonsil hypertrophy [2]. Evidence of tonsil biofilms was found in 11 of the 15 infected specimens studied, with the authors concluding that the tonsillar crypts are frequently sources of large, mixed colonies of bacteria that provide a reservoir of recurrent tonsillitis infections. Interestingly, biofilms were also demonstrated in non-infected tonsil specimens, suggesting that, in addition to being a chronic source of infection, biofilms may form part of the normal bacterial architecture of the tonsils. This raises questions regarding the structure of biofilms themselves, in particular, what microscopic features lead to the development of chronic tonsillitis. It has been demonstrated that biofilms exist in patients who suffer from recurrent tonsillitis. However, their role is not well understood in the context of disease physiology. The only definitive method for treating tonsillitis is tonsillectomy; patients who experience seven episodes of tonsillitis a year warrant tonsillectomy [3]. Despite such guidelines, recent evidence has demonstrated that patients can experience 27 episodes of tonsillitis over 7 years before undergoing tonsillectomy, with considerable burden to the patient and health care services. Furthermore, the operation itself is associated with potentially significant morbidity, with 20% of patients readmitted with bleeding after their operation [4]. Antimicrobial resistance (AMR) is a global challenge. The World Health Organisation has endorsed a global action plan on AMR, and studies have predicted that by 2050, AMR will result in 10-million deaths [5]. To this end, antibiotic stewardship is a key policy within the NHS, with the decision to prescribe antibiotics for recurring tonsillitis being a frequent clinical dilemma.

We hypothesise that the variation of the microbiome in tonsils is associated with the severity of disease and treatment failure with antibiotics. New methodologies and resources of disease models are required to allow an increased understanding of the disease physiology and microbiology of recurrent tonsillitis, as well as the role of biofilms in this process. We need advances in our knowledge of the microbial evolution of tonsillitis to understand the microbial virulence factors in biofilm formation, how this correlates with antibiotic exposure, and the presence of microbes individually or in combination. A 3D tonsil model with mapping data would allow for the investigation of spatial variation in the microbes, microbial metabolism, biofilm formation, and xenobiotic assessment. We need a paradigm shift in research methods for tonsillitis that better represent the clinical condition that we see so frequently. Three-dimensional bioprinting has developed as an advanced technique for depositing living cells and biomaterials to build complex tissue constructs [6,7,8,9,10]. This led to the development and formation of 3D bioprinted biofilms [11,12,13,14,15] as in vitro models for measuring the responses to antibiotic drugs and drug penetration. This new 3D bioprinting bacterial biofilm has several advantages: the embedded bacteria have been shown to possess increased metabolic activity, antimicrobial resistance, and plasmid stability in comparison to traditional 2D models. Therefore, the 3D biofilm potentially mirrors in vivo bacterial growth more closely than 2D models [11,16].

The ideal bioink for this application of tissue engineering has physicochemical properties that permit 3D printing and chemical and mechanical properties that are similar to the desired tissue. Hydrogels are water-based gels composed of polymers dissolved in water and crosslinked to form a solid. There is typically a very high water content with a low mass fraction of polymers. This results in a soft, deformable gel with a highly swollen 3D environment similar to soft tissues. The hydrogel matrix provides an extracellular matrix-like environment for the printed cells and can replicate the mechanical and structural properties of the microenvironment. This is similar to native tissue and supports cells to remain viable and proliferate. This makes hydrogel-based bioinks the most promising candidate for cell carrier and tissue construct fabrication [17]. Different types of bioink may be fabricated to achieve a variety of characteristics. In cartilage tissue engineering, alginate with nanofibrillated cellulose has been utilised as structural bioink to print with fidelity and stability. For tubular structures, pluronics have been utilised as sacrificial bioink to fabricate a hollow structure by adjusting the temperature. In this study, a functional bioink is composited to support biofilm formation. Naturally derived hydrogels like gelatin have superior bioactivity and chemical similarity to the extracellular matrix of natural tissue. This provides a suitable microenvironment for cell adhesion, migration, proliferation, and differentiation [18]. The pitfalls of gelatin hydrogels are limited mechanical strength and biodegradation, which limit application in bioprinting [17]. Synthetic hydrogels like PEGDA (polyethylene glycol diacrylate) offer the capability for photopolymerisation, which provides adjustable mechanical properties and an enhanced control of scaffold architecture during 3D printing [19,20,21,22,23,24]. Synthetic and alginate-based hydrogels lack cellular binding sites, and this limits cell infiltration and growth. Hybrid bioinks, derived from a mixture of natural and synthetic hydrogels, have been developed to overcome the limitations of single-ingredient bioinks. A gelatin-PEGDA hydrogel, for example, combines the biocompatibility of gelatin with the capabilities of PEGDA for photopolymerisation, adjustable mechanical properties, and enhanced control of scaffold architecture during 3D printing.

Three-dimensional printing technologies are useful in the fabrication of tissue scaffolds because they provide precise control of structure and composition. Three-dimensional bioprinting offers high-throughput capability for developing 3D tissue and biofilm constructs [17]. This provides this technology a realistic chance of being applied in the clinical setting for fabricating microbiological models and being more representative of in vivo bacterial growth. Light-based stereolithography (SLA) printing is a novel technology that can fabricate materials with nanometre resolution much quicker than extrusion-based technologies [19]. These printers work by projecting a 2D light pattern through a photocrosslinkable fluid onto a print head. The fluid undergoes photopolymerisation, solidifies, and is fixed to the print head, which is moved upwards through the z plane to construct the 3D shape. Photoabsorbers like tartrazine may be added to improve the printability of internal structures by minimising light scattering and preventing the curing of non-printed areas [25]. Tartrazine is a biocompatible yellow azo dye with a high absorbance at 405 nm wavelength light [19]. Light-based SLA printing is ideally suited for small-scale, precise hydrogel structures with high-resolution channels or perforations [19].

To better understand acute recurrent tonsillitis, we need to move away from studying planktonic cultures and instead focus on understanding surface-associated biofilms. To our best knowledge, this is the first study to develop a 3D-printed gelatin-PEGDA composite hydrogel bioink for the formation of an in vitro tonsillar biofilm.

## 2. Materials and Methods

### 2.1. Bacterial Strains, Growth Media, and Inoculum Preparation

*Pseudomonas fluorescens* (*P. fluorescens*) on nutrient agar (Darwin Biological, Shropshire, UK) was selected for its ability to grow aerobically and anaerobically. It is commensal to humans and generally not clinically significant in human infection [26]. This non-pathogenicity facilitated handling and bioprinting. *P. fluorescens* was inoculated in 100 mL of Lysogeny Broth (LB) (Thermo Scientific, Heysham, UK) and incubated at 37 °C for 18 h (Panasonic MCO-230AIC-PE, Etten-Leur, The Netherlands). The broths were centrifuged in a Heraeus Labofuge 400R at 4300 rpm for 10 min (at 20 °C). The cell pellet was re-suspended in 100 mL phosphate-buffered saline (PBS) (Thermo Scientific, Heysham, UK) and serially diluted to 10^3^ and 10^8^ colony-forming units per milliliter (CFU/mL).

### 2.2. Construct Design

**Figure 1:**
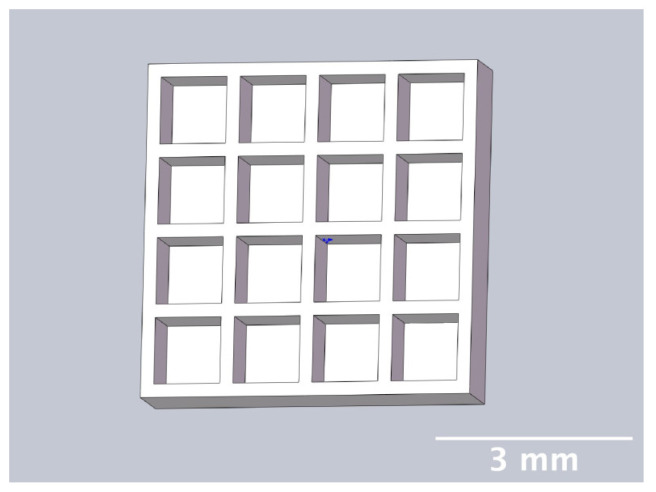
Chitubox screengrab of 5 mm × 5 mm × 1 mm square lattice model with 16 square-shaped pores 800 μm deep 200 μm interstitial wall space (oblique view). This construct was designed to provide large surface area and both oxygenated (thin-walled) and oxygen-depleted (thick-walled) areas for growth of facultative anaerobic bacteria, such as *P. fluorescens*.

Figure 1 shows the designed construct: It is a 5 × 5 × 1 mm lattice with square pores 0.8 mm deep and 0.2 mm thick interstitial wall space created in Solidworks 2022 (Dassault Systèmes, Vélizy–Villacoublay, France).

### 2.3. Hydrogel Development (Biocompatibility and Printability)

To assess biocompatibility and printability of different gelatin-PEGDA hydrogel formulations, four novel hydrogels (gelatin concentrations 0%, 1%, 3%, 5%) were made. A stock gelatin solution was prepared by dissolving 1 g of gelatin powder (Sigma–Aldrich, St. Louis, MO, USA) in 10 mL of deionized water using a magnetic stirrer with a heating plate (Starlab, Milton Keynes, UK) with the temperature set to 38 °C and stir set to 300 spins per minute. This gelatin stock was then added to a solution of tartrazine (89%, pure) (Thermo Fisher Scientific, Loughborough, UK) and lithium phenyl-2,4,6-trimethylbenzoylphosphinate (LAP) (Sigma–Aldrich, St. Louis, MO, USA) dissolved in polyethylene glycol diacrylate (PEGDA) (Sigma–Aldrich, Tokyo, Japan) and deionized water. The weight-per-volume (*w*/*v*) ratios for each gelatin concentration bioink can be seen in Table 1.

For biocompatibility assessment, 20 μL of the 0%, 1%, 3%, and 5% gelatin hydrogel mixtures were placed into separate wells of a Corning 96-well microtiter plate (Sigma–Aldrich, Dorset, UK). Additionally, 20 μL of Lysogeny broth (LB) and 20 μL of *P. fluorescens* suspended in phosphate-buffered saline (PBS) at seeding densities of 10^8^ CFU/mL or 10^3^ CFU/mL were added. Triplicate samples of each gelatin concentration with each of the two seeding densities were made (*n* = 24). Each well sample was then mixed using a separate inoculation loop (Microspec Sterile Plastic Inoculation Loops 5 μL, Fisher Scientific, Loughborough, UK). These hydrogel mixtures were crosslinked using 405 nm light (SUN6 PINK 48 W Nail Lamp UV/LED, SUNUV, Miami, FL, USA) for 120 s then incubated at 37 °C for 9 days. Visual assessment of bacterial growth was performed using images recorded at 4× magnification (Brunei Digital Microscope, Wiltshire, UK) on Days 0, 3, 5, 7, and 9. Optical density of bacterial growth was assessed using a Multiskan Go Microplate Spectrophotometer (Fisher Scientific, Loughborough, UK) at 600 nm absorbance. Statistical analysis was performed using Minitab Statistical Software (v19) [27]. Results are presented as mean value of triplet ± one standard deviation. Absorbance analysis was conducted by paired *t*-test at 95% confidence interval and significance threshold of *p* < 0.05. All results were graphed using Origin 2019b Graphing and Analysis software.

**Figure 2:**
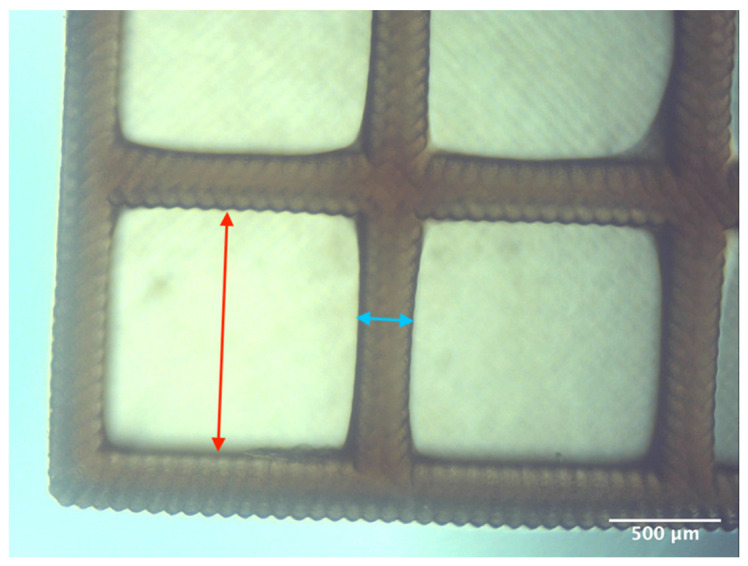
Digital microscope image at 4× magnification demonstrating pore length measurement (red, 1000 μm) and inter-pore distance (blue, 200 μm) (Brunei digital microscope, Wiltshire, UK).

Printability assessment was performed to determine optimum gelatin concentration and 405 nm light exposure time. The printability of each of the 1%, 3%, and 5% gelatin concentration bioinks was tested by printing a lattice design (Figure 1) using the LumenX+ 3D Bioprinter (CELLINK, BICO, Gothenburg, Sweden). Three lattice constructs were printed for each of three gelatin concentrations (1%, 3%, 5%) and rinsed with deionized water. Figure 2 shows a digital microscopic image of a lattice construct marked using Image J software (Version 1.53, National Institutes of Health, Bethesda, MD, USA) to demonstrate pore length and inter-pore distance in μm. Printability was calculated using Equation (1) where *P* is the freehand perimeter (μm) of the pore measured using ImageJ software (version 1.53) and A is the intended area (μm^2^) by design. In this study, A = 10^6^ μm^2^

(1) $$Printability=\frac{P^{2}}{16A}\;$$

**Figure 3:**
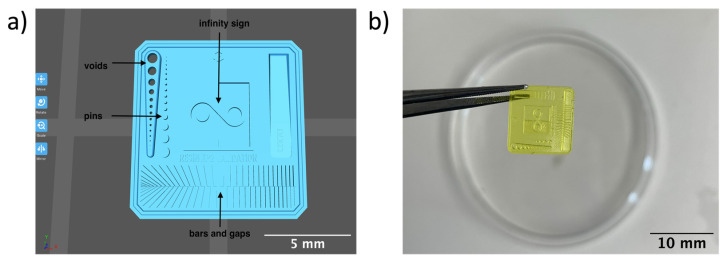
To test the printability of gelatin-PEGDA hydrogel, a designed model containing several validation shapes was printed: (**a**) labelled Chitubox screengrab of XP2 Validation Matrix Model (modified from 3D Printerly, 2023); (**b**) the printed Validation Matrix Model.

Optimum print exposure time of the 1% gelatin ink was assessed using the XP2 validation matrix model (3D Printerly, 2023) (Figure 3). The model was printed at different exposure times (8 s, 8.5 s, 8.75 s, and 9 s), rinsed with deionized water, and inspected under a digital microscope. Print exposure was assessed according to the parameters shown in Table 2.

### 2.4. Bacteria-Laden Bioink Preparation

**Figure 4:**
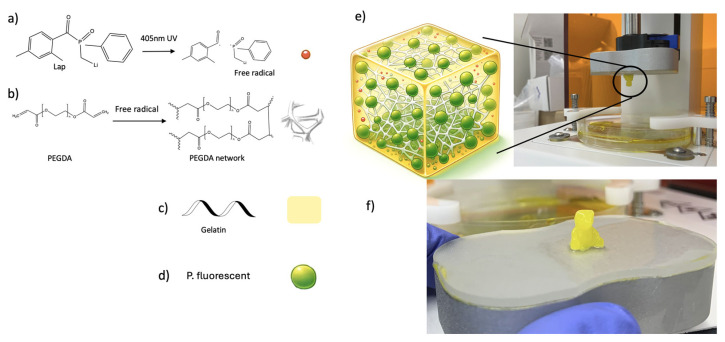
Biofabrication of the gelatin-PEGDA bioink; (**a**) activation of LAP by UV light at 405 nm to generate free radicals (red sphere); (**b**) free radicals react with acrylate groups to induce polymerization of PEGDA, and PEGDA molecules crosslink to form a polymer network (white matrix); (**c**) gelatin forms the basic matrix of the hydrogel with tartrazine (yellow dye); (**d**) *P. fluorescens* (green sphere) harvested from Lysogeny broth was encapsuled in gelatin-PEGDA hydrogel; (**e**) this interpenetrating gelatin-PEDGA hydrogel was printed by stereolithography; (**f**) a designed teddy bear-shaped bacterial-laden hydrogel printed with high resolution.

The hydrogel development tests indicated that the optimal bioink gelatin concentration for printing the construct was 1%. The weight-per-volume (*w*/*v*) ratio for the 1% gelatin concentration bioink was 10% *w*/*v* PEGDA, 1% *w*/*v* Gelatin, 1% *w*/*v* LAP, 88% *w*/*v* DI water, 0.5 mg/mL tartrazine. To make the final bioink, 1 mL of *P. fluorescens* (10^8^ CFU/mL) suspended in PBS, 1 mL of the 1% gelatin hydrogel formulation, and 1 mL of LB were vortex-mixed until homogeneity was achieved, then mixed for a further 30 min in a heated ultrasonic bath. This was repeated for the 0% gelatin hydrogel control. Each ink was stored in an incubator set to 37 °C until immediately prior to bioprinting. Biofabrication of this ink is demonstrated in Figure 4.

### 2.5. Bioprinting and Culture

The Lumen X+ 3D Bioprinter (CELLINK^®^ BICO, Gothenburg, Sweden) was used for all experiments with the following specifications: light wavelength λ = 405 nm (violet), pixel resolution 50 μm, *z*-axis precision 5 μm, projected image 1280 × 800 pixels, layer maximum build volume 64 × 40 × 50 mm, and adjustable print bed heating between 30 °C and 75 °C. The lattice construct (Figure 1) was printed using the Lumen X+ 3D Bioprinter using the printer settings: light power 50% (21.5 mW/cm^2^), exposure time per layer 8.75 s, layer thickness 100 μm, burn-in time factor ×1, and print bed heating 38 °C. All printing was conducted in a Class II microbiology safety cabinet (HERASAFE 2030i, Fisher Scientific, Loughborough, UK) to maintain sterility.

Three constructs for each othe 1% and 0% (control) gelatin concentrations were printed. The constructs were removed from the print head and rinsed with sterile LB before transfer to a sterile Corning 12-well microtiter plate (Sigma–Aldrich, Dorset, UK) for incubation. The cultures were performed at 37 °C in a CO_2_ incubator and LB media were replenished every 2–3 days.

### 2.6. Live/Dead Analysis

Fluorescence staining for live/dead analysis was performed on all printed bacteria-laden constructs after 12 days of incubation. Fluorescein diacetate (FDA) and propidium iodide (PI) stains were used for live and dead cells, respectively, and processed using standard protocol. A ZOETM fluorescent cell imager (Bio–Rad, Hercules, CA, USA) was used for image capture using the following settings: gain = 24, exposure (ms) = 580, LED intensity = 26, and contrast = 24. Live and dead images were merged using Image J software.

### 2.7. Mechanical Characterisation

The mechanical characteristics of hydrogel were evaluated by assessment of compression modulus, swelling ratio, bicinchoninic acid (BCA) assay, and degradation time. For the unconfined compression test, cylindrical molds (8 mm in diameter and 2 mm thick) were used, printed with 8.75 s exposure time and 50% exposure power by the Lumen X+ 183 3D Bioprinter (CELLINK, BICO, San Diego, CA, USA). These cylindrical discs were then soaked in PBS solution at 37 °C for 24 h to achieve swelling equilibrium. Afterward, the hydrogel samples underwent the unconfined compression test. This test was conducted at a ramp velocity of 0.1% of the thickness per second, using the Mechanical Tester model Mach-1™ V500c equipped with a single-axis 250 N load cell. The compressive modulus was calculated from the linear portion of the stress–strain curve, specifically within the 50–100% strain range.

Next, the swelling ratio of four groups of hydrogels was assessed. The hydrogels were printed to a dimension of 5 mm × 5 mm × 3 mm using the LumenX+ 183 3D Bioprinter (CELLINK, BICO, San Diego, CA, USA) with 8.75 s exposure time and 50% exposure power. The swelling behavior was investigated by weighing the samples before (Wo) and after (Wt) immersion in PBS solution at 37 °C at various time points. The swelling ratio was calculated using the Equation (2). After this, BCA assay was used to quantify gelatin release into the buffer at various time points. The concentration of gelatin in the buffer was determined using the Bicinchoninic Acid (Micro BCA Protein Assay Kit, Thermo Scientific). A standard curve was established using gelatin solutions in PBS at varying concentrations (200, 40, 20, 10, 5, 2.5, 1, 0.5 μg/mL), described by the quadratic equation Y = −2.587 + 32.39 × X + 76.79 × X^2^, R^2^ = 0.998. At each time point, the buffer samples collected from each experimental group were mixed with the working reagent provided in the BCA assay kit and subsequently incubated for 2 h. Following this incubation period, the samples were cooled down to room temperature. The absorbance of these samples was then measured at 562 nm using a spectrophotometric plate reader. The gelatin concentration in each sample was determined by referencing these absorbance values against the established standard curve. The Gelatin release was calculated by Equation (3), where “Wgelatin detected” represents the weight of gelatin detected in BCA assay, and “Wgelatin printed” represents the weight of gelatin printed in the sample.

Finally, evaluation of hydrogel degradation was conducted by culturing the lattice structures in PBS in an incubator over specific time intervals of 1, 3, and 5 days. To accurately assess the structural stability and integrity of the hydrogels over these periods, detailed microscopic observations were performed. Lattice structures were observed by microscopy at 4× magnification. This approach provided visual evidence of any structural changes or degradation occurring within the hydrogel matrices. To test the mass loss during the degradation in PBS solution, samples from four groups of hydrogels were assessed. The hydrogels were printed to a dimension of 5 mm × 5 mm × 3 mm using the Lumen X+ 183 3D Bioprinter (CELLINK, BICO, Gothenburg, Sweden) with 8.75 s exposure time and 50% exposure power. Then, the samples were weighed (*Wi*) and incubated in 2 mL PBS solution, and the solution was changed every other day. At each time point, the samples were lyophilized and weighted (*Wd*). The mass loss percentage was calculated by Equation (4).

(2) $$Swelling\;ratio\;\%=\frac{Wt-Wo}{Wo}\;\%$$

(3) $$Gelatin\;release\;\%=\frac{W_{gelatin\;detected}\;}{W_{gelatin\;printed}}\;\%$$

(4) $$Mass\;loss\;\%=\frac{Wi-Wd}{Wi}\;\%$$

All tests were performed in triplicate. For statistical analysis, a one-way ANOVA followed by a normality test was applied. A *p*-value of less than 0.05 was considered statistically significant.

## 3. Results

### 3.1. Hydrogel Printability

Table 3 summarises the printability of each gelatin concentration bioink (1%, 3%, and 5%). Mean values (*n* = 3 ± one standard deviation) for the pore length, the distance between the pores, and the printability (Equation (1)) are shown. Table 4 demonstrates the optimal exposure time of the 1% gelatin bioink to be 8.75 s, where superiority is defined as equal numbers of pins and voids and symmetrical continuity of the Infinity sign point.

### 3.2. Hydrogel Biocompatibility

**Figure 5:**
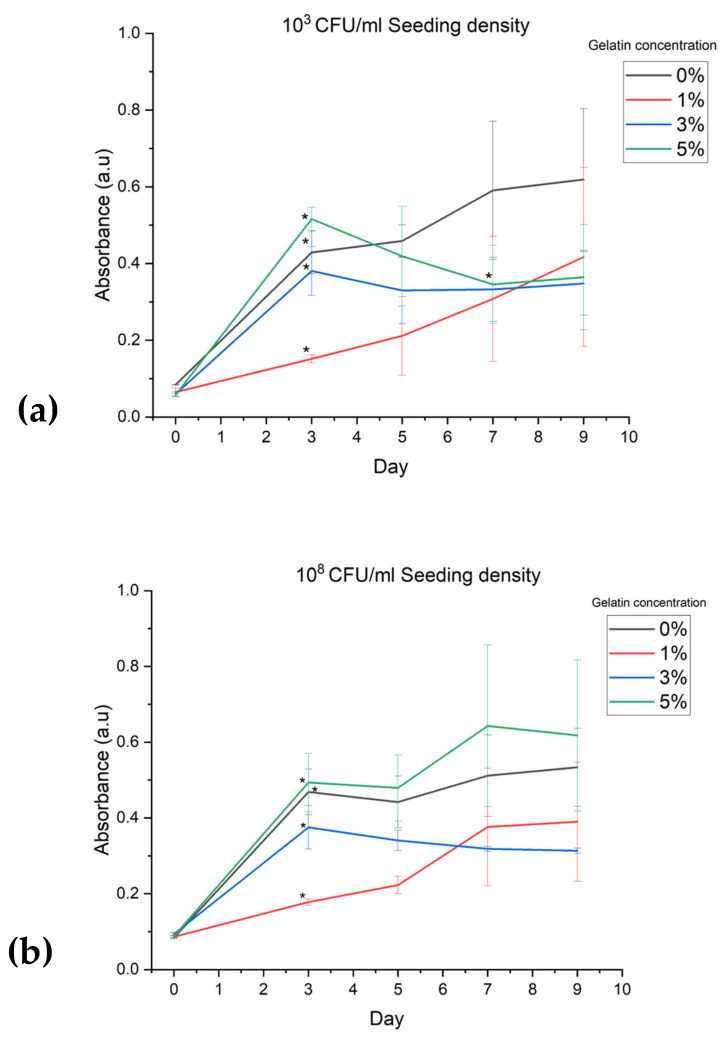
Absorbance data for four gelatin-PEGDA hydrogel formulations (0%, 1%, 3%, and 5%) seeded at bacterial densities of (**a**) 10^3^ CFU/mL and (**b**) 10^8^ CFU/mL. Absorbance at 600 nm was used to quantify optical density of bacterial growth, where greater absorbance suggests greater density of bacteria growth. Data are presented as mean value ± one standard deviation, with asterisk (*) denoting significant difference between Day 0 and Day 3 of culture (paired *t*-test, 95% confidence interval).

Absorbance analysis of the optical density of the hydrogels was used to determine bacterial growth on Day 0, 3, 5, 7, and 9 of a 9-day incubation period. Figure 5a,b shows absorbance data for four different concentrations (0%, 1%, 3%, and 5%) of gelatin hydrogel mixtures with 10^3^ CFU/mL and 10^8^ CFU/mL *P. fluorescens*, respectively.

Figure 5a shows that absorbance of all concentrations of gelatin increased significantly from Day 0 to Day 3 for the 10^3^ CFU/mL *P. fluorescens* seeding density (0%, *p* = 0.008; 1%, *p* = 0.020; 3%, *p* = 0.013; 5%, *p* = 0.002). The 0% gelatin hydrogel demonstrated the greatest absorbance reading at Day 9. The 1% gelatin hydrogel had continuous and consistent upwards growth compared with all other gelatin concentrations over the 9 days and did not decline or plateau. Absorbance in the 3% and 5% gels faltered after Day 5. Figure 5b shows that absorbance of all concentrations of gelatin increased significantly from Day 0 to Day 3 for the 10^8^ CFU/mL *P. fluorescens* seeding density (0%, *p* = 0.008; 1%, *p* = 0.004; 3%, *p* = 0.012; 5%, *p* = 0.011). The 5% gelatin hydrogel demonstrated the greatest absorbance reading at Day 9. The 1% gelatin concentration displayed steadily increasing absorbance before plateau between Day 7 and Day 9. The 3% gelatin hydrogel faltered after Day 3.

**Figure 6:**
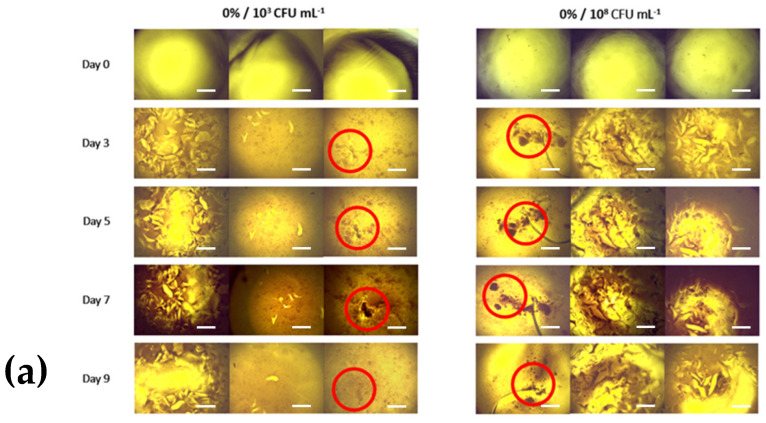

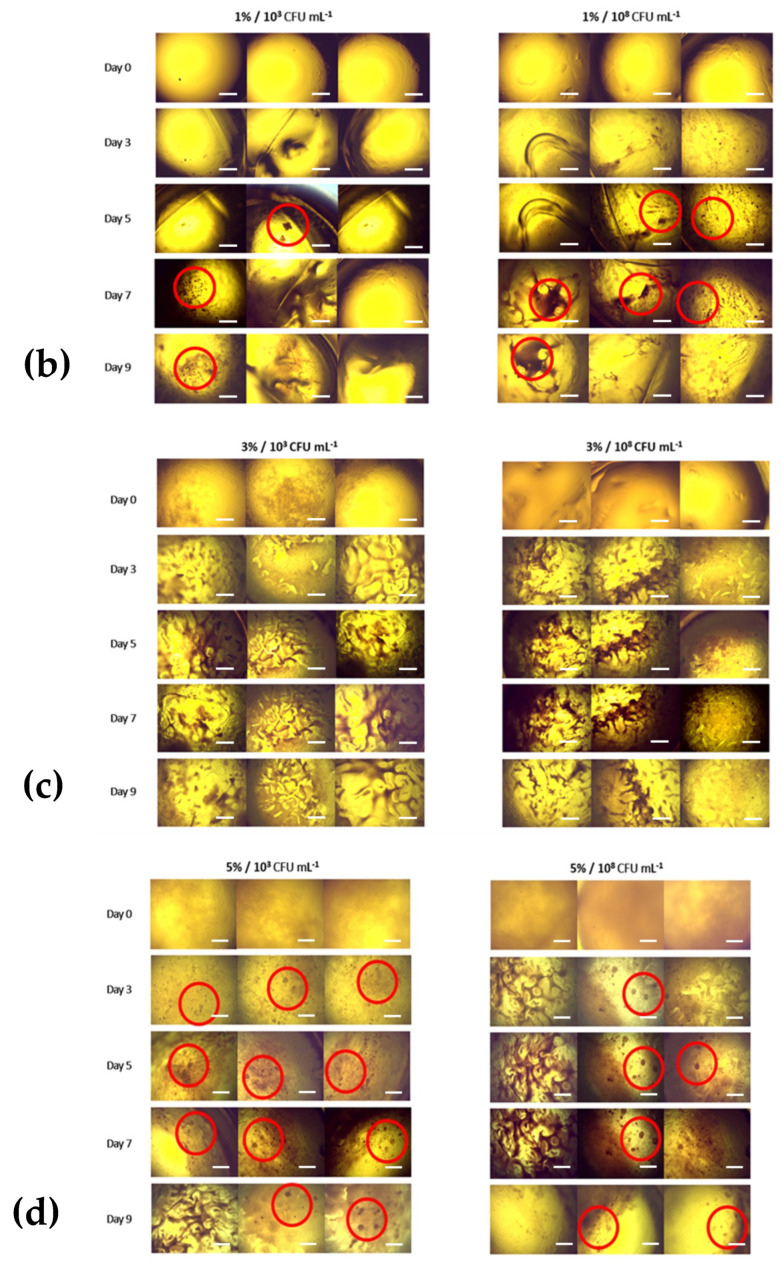
Triplicate digital microscope images of (**a**) 0% (**b**) 1% (**c**) 3% (**d**) 5% *w*/*v* gelatin-PEGDA hydrogels at seeding densities of 10^3^ and 10^8^ CF/mL and Days 0, 3, 5, 7, and 9 of culture (10× magnification). Bacterial aggregates with morphology consistent with biofilm formation are highlighted in red. Scale bar is 250 µm.

Digital microscopy images were recorded on Days 0, 3, 5, 7, and 9 for a visual inspection of bacterial growth. Images are presented below for each of the 0%, 1%, 3%, and 5% hydrogels and 10^3^ CFU/mL and 10^8^ CFU/mL seeding density of *P. fluorescens*, in triplicate (Figure 6). Figure 6a demonstrates an increased optical density of the 10^3^ CFU/mL seeded hydrogels compared to the 10^8^ CFU/mL hydrogels, suggested by a comparatively dull appearance. In the 1% gel (Figure 6b), bacterial colonies are seen more in the 10^8^ CFU/mL seeded gel than in the 10^3^ CFU/mL seeded gel. In the 3% gel (Figure 6c), there are no larger bacterial aggregates and no apparent differences between the two seeding densities. Both seeding densities of 5% gelatin hydrogel mixtures appear to have had considerable bacterial growth (Figure 6d).

### 3.3. Live/Dead Analysis

**Figure 7:**
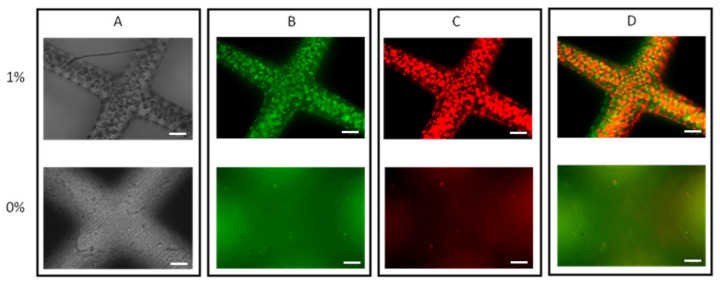
Fluorescent microscopy images of 0% and 1% *w*/*v* gelatin-PEGDA hydrogel formulations after PI/FDA staining (10× magnification). (**A**) Brightfield images; (**B**) live-stain images (FDA); (**C**) dead-stain images (PI); (**D**) Merged live- and dead-stain images. Scale bar is 100 µm.

Bacterial viability is demonstrated in the 1% gelatin hydrogel but not the 0% hydrogel (Figure 7).

### 3.4. Mechanical Characterisation of Hydrogel

Compression testing, swelling ratio, BCA assay, and degradation analysis were performed.

## 4. Discussion

### 4.1. Approach

Acute recurrent tonsillitis is a chronic, biofilm-related infection that is a significant burden to patients and healthcare systems and contributes to antimicrobial resistance. Biofilm models are key to improving the understanding of acute recurrent tonsillitis and other chronic biofilm-related infections. They are likely to be valuable in the development of adjunctive therapies targeting antibiotic persistence, especially in the context of increasing antimicrobial resistance. The most promising technology for the fabrication of biofilm models is the 3D printing of hydrogels. This is the only technique with which a full in vitro biofilm life cycle has been demonstrated [11]. Stable, mature, in vitro biofilms offer the closest representation of chronic in vivo infections. Cellular viability analysis has been established as a useful tool for the evaluation of in vitro biofilms. This technique is used to assess whether printed cells are viable and whether they undergo the necessary physiological cues to assume the biofilm phenotype [11]. The primary aim of this study was to develop a proof-of-concept construct design, as well as bioink and printing methods that facilitate the assessment of 3D bacterial growth in a hydrogel model. A novel PEGDA–gelatin bioink was successfully inoculated with bacteria and bioprinted, cultured, and demonstrated bacterial growth within a newly developed construct design.

### 4.2. Construct Design

A lattice construct used in this study was adapted from previous constructs that had been proven to be successful in growing mature biofilms [11]. The surface area of the construct was maximized by increasing the size of the construct and by incorporating partial thickness perforations rather than full thickness. This was intended to provide both oxygenated and oxygen-depleted areas for the growth of facultative anaerobic bacteria, such as *P. fluorescens* and bacterial species implicated in tonsillitis. The interstitial wall thickness of 0.2 mm was intended to promote nutrient diffusion from the media to further enhance bacterial growth.

### 4.3. Hydrogel Development and Bioprinting

Results obtained from the printability test indicated that the optimal bioink gelatin concentration for printing the lattice construct was 1%. The differences between the measured and desired values for the pore length, distance between the pores, and printability were the lowest for the 1% gelatin concentration bioink. Gelatin concentration and bioink viscosity were found to be positively related, and lower concentrations of gelatin resulted in a greater ease of bioprinting. After confirmation of the appropriate gelatin concentration, the printing conditions were optimised by a validation matrix. This model allowed for a calibration of the exposure time to find the optimal settings to provide a high-resolution print using the 1% gelatin concentration. The optimised exposure time of 8.75 s resulted in a short total print time, which allowed for fast biofabrication. This is advantageous for a clinical setting, such as modeling acute recurrent tonsillitis for testing appropriate treatment, as it allows for the possibility of high-throughput testing.

### 4.4. Mechanical Characterisation of Hydrogel

**Figure 8:**
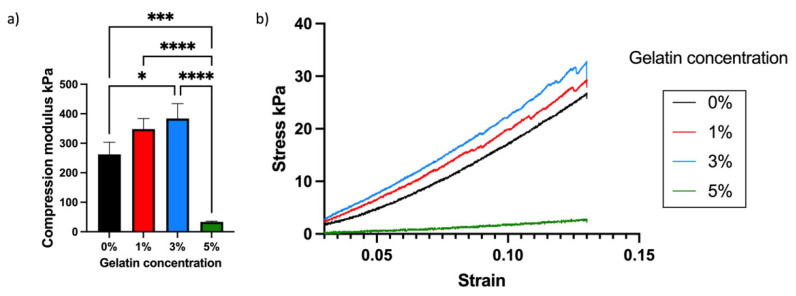
Compression modulus (**a**) and stress–strain curve (**b**) of different gelatin-PEGDA hydrogels, demonstrating significant difference in compression modulus and stress between the 0% and 3% gels, and for the 5% gel in comparison to all other gels. The compression moduli were 262.3 ± 41.3, 348 ± 36.05, 383.9 ± 50.27, and 33.51 ± 2.67 kPa (for 0%, 1%, 3%, and 5% respectively), with asterisks (*, ***, ****) signifying independent significant differences.

**Figure 9:**
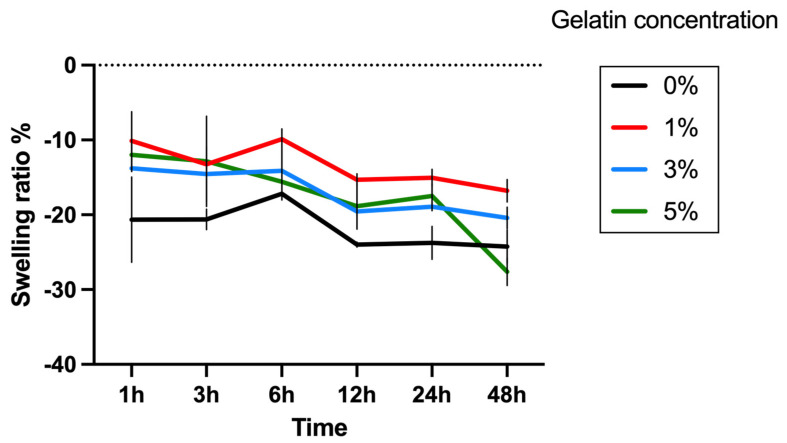
Swelling ratio data. These compare weight of construct at time of fabrication and at different timepoints after immersion in 37 °C PBS. All constructs lost mass, with the 1% gel demonstrating least loss and the 5% gel most loss.

**Figure 10:**
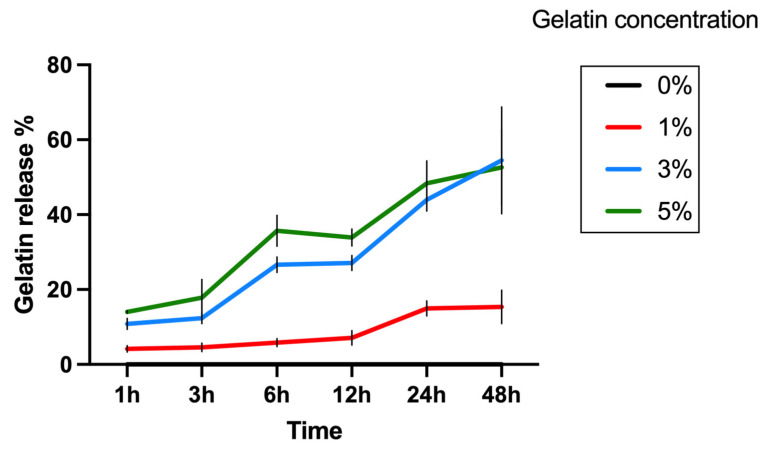
Gelatin release as a percentage of gelatin detected by BCA assay compared to mass of gelatin printed for each hydrogel formulation. The 1% gel demonstrated far less gelatin release compared to the 3% and 5% gels. The 0% gel was not included in this assay as it does not contain any gelatin.

**Figure 11:**
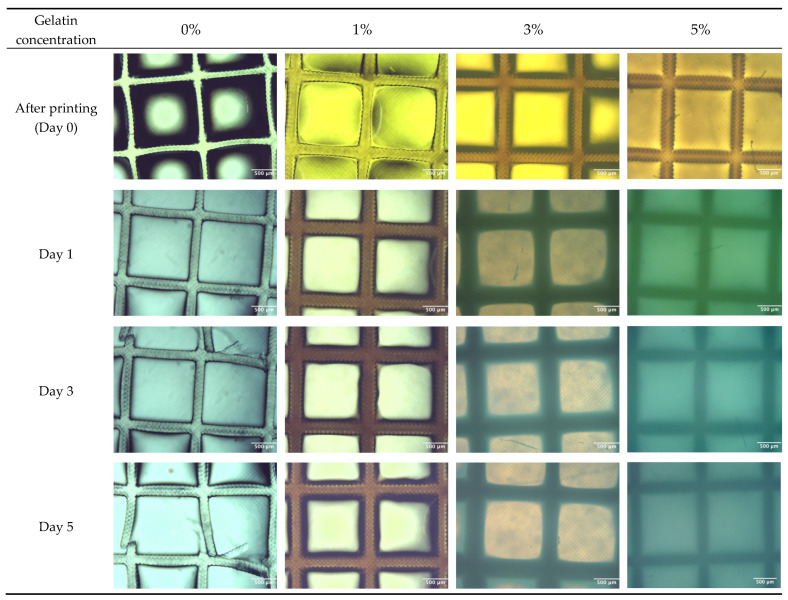
Microscopic images at 4× magnification of constructs at 0, 1, 3, and 5 days. The 0% gel shows poorer preservation of structure in comparison to the 1%, 3%, and 5% gels.

**Figure 12:**
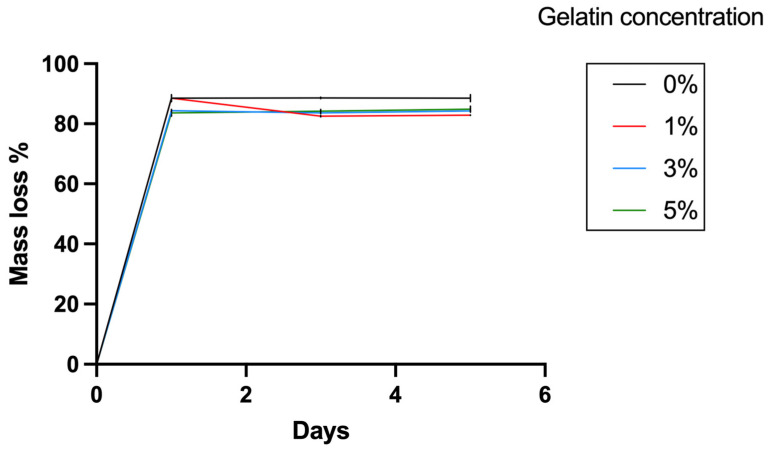
Percentage mass loss of hydrogels at 0–5 days. The mass loss after lyophilization is around 80%, reflecting the high water content with a low mass fraction of polymer found in hydrogels. The 0% gelatin gels demonstrated slightly greater percentage of mass loss than the gels with gelatin.

A variation in visco-elastic properties of gelatin-PEGDA hydrogels, depending on the component ratio, has been reported previously [28]. In the present study, the compression modulus was significantly greater in the 3% compared to the 0% gel, implying that the addition of gelatin strengthened the gel (Figure 8). It is hypothesised that the significantly reduced modulus in the 5% gel (Figure 9) was due to the swelling of uncrosslinked gelatin disrupting the structure after immersion in PBS. The 1% gel demonstrated the least percentage mass loss, suggesting superior preservation of the hydrogel matrix (Figure 10). The presence of uncrosslinked gelatin is consistent with the comparatively greater gelatin release seen in the 5% gel as measured by the BCA assay (Figure 11). Degradation testing (Figure 12) demonstrated a structural failure of the 0% gel, but the favoured 1% gel was able to maintain structure during incubation in PBS. We conclude that the addition of between 1% and 3% gelatin to PEGDA is sufficient to improve the compression modulus, swelling ratio, gelatin release, and degradation. In summary, the addition of gelatin enhances the mechanical properties of PEGDA hydrogels.

### 4.5. Hydrogel Biocompatibility

The assessment of biocompatibility was conducted by spectrophotometry using absorbance readings recorded after incubation. The optical density of all gelatin concentrations and seeding densities significantly increased from Day 0 to Day 9 (Figure 4a,b). In hydrogels seeded at 10^3^ CFU/mL (Figure 6a), growth in the 3% and 5% gelatin gels faltered after Day 3. The 0% and 1% gels increased throughout the incubation period, with the 0% gel demonstrating the greatest overall absorbance reading after 9 days. In hydrogels seeded at 10^8^ CFU/mL (Figure 6b), a significant increase in absorbance from Day 0 to Day 3 was also seen. The 3% gelatin gel faltered after Day 3. The 0% and 1% gels increased steadily throughout the incubation period, and the 5% gel demonstrated the greatest overall absorbance reading after 9 days. Microscopic changes consistent with bacterial growth were seen in all hydrogel formulations and seeding densities (Figure 5, Figure 6, Figure 7 and Figure 8). There was comparably reduced growth in the 3% and 5% 10^3^ CFU/mL gels on visual inspection (Figure 6 and Figure 7). These data suggest that all novel gelatin-PEGDA hydrogels supported bacterial growth but to different extents. When collagen is hydrolysed to form gelatin, it displays reduced antigenicity but maintains arginyl-glycyl-aspartic acid (RGD) tripeptides for integrin-mediated cell adhesion and matrix metalloproteinase-sensitive degradation sites for enzyme degradation [29]. This makes GelMA gelatin not just biocompatible, but bioactive in the essential cellular functions of attachment, migration, and proliferation [29]. It was, therefore, hypothesised that greater bacterial growth would be observed in hydrogels with a greater percentage *w*/*v* of gelatin, but this was not observed. When increasing gelatin concentration, there is an increase in the viscosity of the mixture caused by the intermolecular cross-bonding of gelatin molecules [30]. This may impair nutrient diffusion, limiting bacterial proliferation. The absorbance behaviour of the 10^8^ CFU/mL gels was similar to the behaviour of the 10^3^ CFU/mL gels. A higher seeding density was chosen to promote accelerated biofilm formation. These data, in addition to those from the printability assessment, supported the use of 1% *w*/*v* gelatin hydrogel seeded with *P. fluorescens* at 10^8^ CFU/mL.

### 4.6. Hydrogel Culture

A bacteria-laden construct was successfully printed and cultured with demonstrated bacterial growth. The printed hydrogel structures maintained mechanical stability after the 12-day incubation period was suspended in LB (Figure 5, Figure 6, Figure 7 and Figure 8). Bacterial growth was observed in all cultured constructs during live-/dead-cell staining (Figure 9). This demonstrated superior bacterial growth in the 1% gelatin gel, both on the surface and within the interstitium of the gel, whereas in the 0% gel, growth was limited to the surface. This was expected as gelatin is a bio-active polymer that provides a microenvironment that supports cell adhesion, migration, proliferation, and differentiation [18]. The replication of the mechanical and structural properties of the cellular microenvironment is lacking in synthetically derived hydrogels, represented here by the 0% gelatin control (PEGDA only). Bacterial growth in the 0% gel was likely surface-associated and reliant on direct contact with the LB medium for nutrition. A formal analysis of biofilm morphology was not performed.

### 4.7. Limitations

The use of non-clinical, non-tonsillar bacteria limits the clinical applicability of this study to proof-of-concept only. The bacterial strain used, pseudomonas fluorescens, was selected specifically for non-pathogenicity, which facilitated the handling and bioprinting. This strain is commensal to humans and generally not clinically significant in human infection [21]. Further study with clinically significant organisms, particularly GAS, is required to create a biofilm model with greater clinical applicability. A 405 nm light SLA printer was used for the primary crosslinking of the hydrogel constructs. This wavelength of light is known to be bactericidal, but the dose of 405 nm light irradiation is lower than previous bacterial eradication studies [31,32], which showed a significant reduction in colony-forming units (CFU) only after >50 min of irradiation at 10 w/cm^2^. Additionally, the sum of multiple repeated low-power exposures experienced by the bacteria during sequential layering in SLA printers has been shown to be equivalent to a single higher-power exposure rather than additive or multiplicative [19]. We hypothesise that tartrazine may be a protective factor. This dye has a high absorbance at 405 nm and is used primarily to reduce light scattering and the curing of non-printed areas. However, this process may also shield bacteria seeded in proximal layers as the sequential layers are irradiated by the SLA printer. Regardless, bacterial growth was observed. This suggests that the presented printing process has an insignificant bactericidal effect. The determination of the role of tartrazine in bacterial protection would require a formal investigation. Construct cultures were only incubated for 12 days, but a previous successful biofilm culture was demonstrated over 28 days [11]. Increased culture duration is recommended to demonstrate mature biofilm formation.

## 5. Conclusions

A light-based 3D bioprinting method was used to demonstrate the fabrication of a bacteria-laden hydrogel structure using a gelatin-PEGDA hybrid ink, with superior bacterial growth after culture for 12 days in comparison to PEGDA hydrogel control. Printability and mechanical testing suggest that a 1% gelatin concentration bioink is the optimal formulation for both bacterial growth and high-resolution printing. The results of this work will support advancements in the biofabrication of biofilm models, leading to an improved understanding of acute recurrent tonsillitis and other chronic biofilm-associated infections. This knowledge could support the development of novel anti-biofilm therapeutics, which are an important adjunct in the fight against antimicrobial resistance. Initiating a study with clinically relevant ex vivo tonsil bacteria will be an important next step in improving the treatment of this prodigious, impactful, but understudied disease.

## Figures and Tables

**Table 1:** Bioink formulations for each gelatin-PEGDA hydrogel used in printability testing.

Gelatin Concentration	Bioink Formulation
5%	10% *w*/*v* PEGDA, 5% *w*/*v* Gelatin, 1% *w*/*v* LAP, 84% *w*/*v* DI water, 0.5 mg/mL tartrazine
3%	10% *w*/*v* PEGDA, 3% *w*/*v* Gelatin, 1% *w*/*v* LAP, 86% *w*/*v* DI water, 0.5 mg/mL tartrazine
1%	10% *w*/*v* PEGDA, 1% *w*/*v* Gelatin, 1% *w*/*v* LAP, 88% *w*/*v* DI water, 0.5 mg/mL tartrazine

**Table 2:** Assessment criteria for determination of optimum 405 nm light exposure time.

	Pins and Voids	Infinity Sign	Bars and Gaps
Under Exposed	Number of pins < Number of voids	Small gap in the middle of the infinity sign	Bars that fit down gaps with extra room
Over Exposed	Number of pins > Number of voids	Small overlap in the middle of the infinity sign	Bars too big to fit down gaps
Correctly Exposed	Number of pins = Number of voids	Middle of the infinity sign just touching	Bars and gaps that are the same size

**Table 3:** Mean pore length, inter-pore distance, and calculated printability value for 1%, 3%, and 5% *w*/*v* gelatin gelatin-PEGDA hydrogels. Values are provided as mean ± one standard deviation. The 1% formulation most closely replicated the desired measurement for all criteria.

	Gelatin Concentration			Measurement
	1%	3%	5%	
Pore Length (μm)	1120 ± 17.8	798 ± 109	874 ± 51.8	1000
Distance Between Pores (μm)	156 ± 57.8	413 ± 84.5	280 ± 19.3	200
Printability	0.998 ± 0.033	1.44 ± 0.054	1.31 ± 0.085	1

**Table 4:** Digital microscopic images of 1% gelatin-PEGDA hydrogel 3D-printed XP2 validation matrix (Figure 3). Exposure time of 8.75 s provides superior symmetry in comparison to other tested durations.

Exposure Time (Seconds)	Number of Pins and Voids		Infinity Sign	Bars and Gaps	Conclusion
	Pins	Voids			
8	6	10	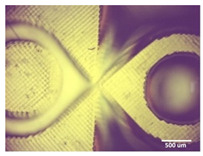	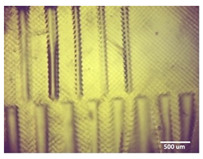	Underexposure
8.5	9	10	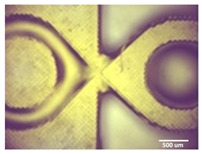	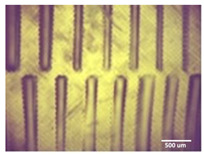	Underexposure
8.75	10	10	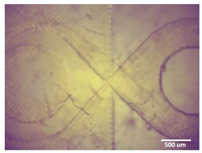	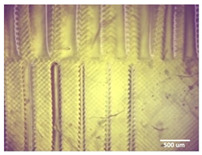	Correct Exposure
9	11	10	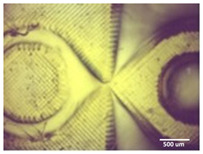	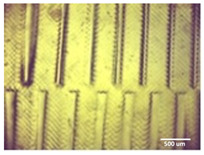	Overexposure

## Data Availability

Data are contained within the article.
